# Chondral injuries in patients with recurrent patellar dislocation: a systematic review

**DOI:** 10.1186/s13018-022-02911-1

**Published:** 2022-01-31

**Authors:** Filippo Migliorini, Emanuela Marsilio, Francesco Oliva, Jörg Eschweiler, Frank Hildebrand, Nicola Maffulli

**Affiliations:** 1 Department of Orthopedic, Trauma, and Reconstructive Surgery, RWTH Aachen University Hospital, Pauwelsstraße 30, 52074 Aachen, Germany; 2 Department of Musculoskeletal Disorders, Faculty of Medicine and Surgery, University of Salerno, Via S. Allende, SA 84081 Baronissi, Italy; 3 School of Pharmacy and Bioengineering, Faculty of Medicine, Keele University, Thornburrow Drive, Stoke on Trent, England; 4 Barts and the London School of Medicine and Dentistry, Centre for Sports and Exercise Medicine, Mile End Hospital, Queen Mary University of London, 275 Bancroft Road, London, E1 4DG England

**Keywords:** Patellar dislocation, Chondral damage, Soft tissues

## Abstract

**Background:**

Patellar dislocations in patients presenting with recurrent patellofemoral instability can damage the surrounding structures, limiting patient’s participation to recreational activities and quality of life. This study evaluated frequency, location, and extent of associated injuries in patients with recurrent patellar dislocation.

**Methods:**

This systematic review was conducted according to the PRISMA checklist. PubMed, Google scholar, Embase, and Web of Science databases were accessed in July 2021. All the published clinical studies reporting frequency, location, and extent of soft tissue lesions in patients with recurrent patellar dislocations were accessed.

**Results:**

Data from 9 articles (232 patients) were retrieved. The mean age of the included patients was 21.2 ± 5.6 years. 84.8% of patients suffering from recurrent patellar dislocations demonstrated patellar chondral defects: medial facet (34.9%), while patellar crest (34.8%) and lateral facet (17%). 27.8% of patients demonstrated trochlear chondral injuries.

**Conclusion:**

Chondral defects of the medial facet and the crest of the patella are the most common in patients with recurrent patellofemoral instability.

## Introduction

Recurrent patellofemoral instability is common, especially among the active and young population [1, 2]. Its aetiogenesis is multifactorial [3, 4]. Several pathoanatomical factors which predispose to instability have been described, such as patella alta, dysplasia, mal-alignment syndromes, and leg axis deformities [5–9]. Irrespective of the aetiopathogenesis, most of patients experience recurrent episodes of patellar dislocation [10, 11]. Recurrent patellar dislocations may damage the articular surface, generating chondral or osteochondral defects [12]. Chondral injuries may cause persistent pain, limiting knee function and impairing the patients’ quality of life [13–15]. Controversies regarding the frequency, extent, and location of chondral lesions exist [16–18]. Previous studies reported that many patients with recurrent patellofemoral instability evidenced chondral defects and osteochondral fractures on the medial facet of the patella and on the lateral trochlea [19–21]. However, the evidence with regard of frequency, location, and extent chondral damages in patients with recurrent patellofemoral instability are limited and no previous systematic review has been published [22–30]. This systematic review evaluated the frequency, location, and extent chondral damages in patients with recurrent patellofemoral instability.

## Material and methods

### Search strategy

This systematic review was conducted according to the Preferred Reporting Items for Systematic Reviews and Meta-Analyses: the PRISMA guidelines [31]. The literature search was guided by the following points:

- Problem: recurrent patellar dislocation;
- Outcome: soft tissue injuries.

### Literature search

Two independent authors (**;**) performed the literature search in July 2021. PubMed, Google Scholar, Embase, and Web of Science were accessed to identify suitable articles. The database search was performed without filters and time constrains, using the Boolean operators AND/OR. The following keywords were used in combination: *patella, dislocation, recurrent, instability, soft tissue, chondral, articular, cartilage, lesion, osteochondral, injury, arthroscopy, medial patellofemoral ligament, MPFL, damage, insertion, rupture, Outerbridge, and International Cartilage Repair Society, ICRS.* The same authors performed the initial screening of the resulting titles from the search in a separate fashion and accessed the full text of the articles of interest. The bibliographies of the full-text articles were screened by hand for identify further eligible articles. Any disagreements were discussed and settled by consensus.

### Eligibility criteria

All the published clinical studies which reported quantitative data on frequency, location, and extent of chondral injuries in patients with recurrent patellar dislocations were considered. Given the authors language capabilities, articles in English, German, Italian, French, and Spanish were eligible. Level I–IV of evidence, according to Oxford Centre of Evidence-Based Medicine [32], was considered. Reviews, technical notes, comments, letters, editorials, protocols, and guidelines were not eligible, nor were biomechanical, animal, and cadaveric studies. Studies reporting data on habitual, congenital, and/or acute patellofemoral instability were excluded. Studies involving patients who underwent previous patellofemoral surgical procedures were also not eligible. Missing information on the endpoints of interest warranted the exclusion from this study.

### Outcomes of interest

Data extraction was performed by two authors (**;**). Studies generalities were collected: author, year, journal, study design, number of patients, and mean age. Arthroscopy findings were also collected: type, location, and extent of trochlear and patellar chondral injuries. The International Cartilage Repair Society (ICRS) [33] was used to classify the arthroscopic findings.

### Methodology quality assessment

Two authors (**;**) independently assessed the methodological quality using the Newcastle–Ottawa Scale (NOS) [34]. NOS was used to assess methodological quality of the included studies. A 'star system' was applied, in which a study is judged on three broad perspectives: the selection of the study groups; the comparability of the groups; and the observation of either the exposure or outcome of interest for case–control or cohort studies respectively. Mean values of 2 stars in selection, 1 or 2 stars in comparability, and 2 or 3 stars in outcomes were considered satisfactory.

## Results

### Search result

**Fig. 1:**
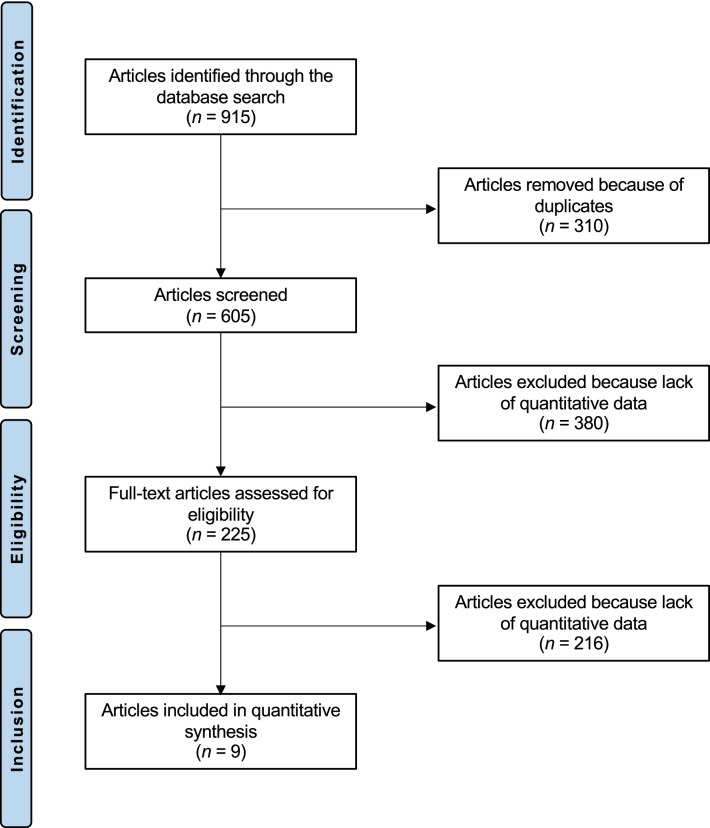
Flowchart of the literature search

The literature search resulted in 915 articles. Of these, 310 were excluded being duplicates. Another 380 were not eligible: not matching the topic (*N* = 220), study design (*N* = 90), acute patellofemoral instability (*N* = 45), language limitation (*N* = 10), and uncertain results (*N* = 15). This left 225 articles for inclusion. A further 216 articles were excluded because lack of data under the outcomes of interest. Finally, 9 articles were considered for the analysis (Fig. 1).

### Methodological quality assessment

The satisfactory size of the included studies, their baseline comparability, and adequate length of the follow-up are the most important strengths of this analysis. The most important limitations evidenced by the NOS are the lack of randomization and blinding, along with high the high risk of bias during allocation concealment. Concluding, the NOS resulted in 3 or 4 stars in selection, 1 or 2 stars in comparability, and 2 or 3 stars in outcomes in all of the selected articles, attesting to this study good quality of the methodological assessment (Table 1).

**Table 1:** Methodological quality assessment

References	Selection	Comparability	Outcome
Boddula et al. [22]	****	**	***
Chan et al. [23]	****	*	**
Franzone et al. [24]	****	**	**
Gaweda et al. [25]	****	**	***
Kita et al. [26]	****	**	***
Lee et al. [27]	****	**	***
Luhmann et al. [28]	****	*	**
Maffulli et al. [29]	****	**	***
Nha et al. [30]	****	**	***

### Patient demographics

A total of 232 patients were identified, with a mean age of 21.2 ± 5.6. Study generalities and patient demographics of the included studies are shown in Table 2.

**Table 2:** Study generalities and patient demographics of the included studies

References	Journal name	Design	Knees	Mean age
Boddula et al. [22]	Am J Sports Med	Retrospective	20	28.0
Chan et al. [23]	Knee Surg Sports Traumatol	Retrospective	1	12.0
Franzone et al. [24]	J Knee Surg	Retrospective	38	21.0
Gawada et al. [25]	Int Orthop	Prospective	19	25.5
Kita et al. [26]	J Orthop Sci	Retrospective	31	20.0
Lee et al. [27]	Knee Surg Sports Traumatol	Retrospective	28	20.0
Luhmann et al. [28]	J Pediatr Orthop	Retrospective	38	14.9
Maffulli et al. [29]	Injury	Prospective	34	25.6
Nha et al. [30]	Am J Sports Med	Retrospective	23	26.0

### Main findings

84.8% of patients demonstrated patellar chondral defects: 34.9% in the medial facet, 17.0% in the lateral facet, and 34.8% in the patellar crest. Concerning the medial facet, defects were ICRS grade I in 11.1%, grade II in 14.8%, grade III in 9.3%, and grade IV in 9.3%. Concerning the lateral facet, defects were ICRS grade I in 9.3%, grade II in 11.1%, grade III in 3.7%, and grade IV in 4.3%. Concerning the patellar crest, defects were ICRS grade I in 9.7%, grade II in 25.8%, grade III in 6.5%, and grade IV in 19.0%. 27.8% of patients demonstrated trochlear chondral defects. These defects were ICRS grade I in 19.2%, grade II in 16.7%, grade III in 5.1%, and grade IV in 7.0%. Table 3 resumes the main findings of the included studies.

**Table 3:** Main findings of the included studies

References	Main findings
Boddula et al. [22]	Seven patients showed chondral lesions, located at medial and lateral patellar facet, while one patient reported an associated lateral trochlea chondral lesion
Chan et al. [23]	Chondral fracture and a lateral trochlea chondral lesion were found in one patient
Franzone et al. [24]	Twenty patients had patellar chondral lesions, while five patients reported lateral trochlea defects
Gaweda et al. [25]	Nineteen patients had severe and extensive patellar chondral defects
Kita et al. [26]	Twenty-seven patients reported patellar lesions, while eight showed lateral trochlea chondral defects, after MPFL rupture
Lee et al. [27]	After MPFL reconstruction, 26 patients presented patellar chondral lesions, while16 patients had lateral trochlea lesions
Luhmann et al. [28]	Patellar osteochondral lesions were present in 30 knees, femoral lesions were documented in 11 knees, and loose bodies were present in 6 knees
Maffulli et al. [29]	At arthroscopy, there was an osteochondral lesion less than 15 mm in diameter in 13 of 34 patients. The osteochondral defects were in the medial patellar facet (*n* = 6), on the lateral femoral trochlea (*n* = 4), and on both the medial patellar facet and the lateral femoral trochlea in 3 patients
Nha et al. [30]	All patients reported patellar lesions, while 14 showed lateral trochlea chondral defects, after MPFL rupture

## Discussion

According to the main findings of the present systematic review, 84.8% of patients suffering from recurrent patellar dislocation demonstrated chondral defects of the patella. Defects are more frequently located on the medial facet (34.9%), while patellar crest (34.8%) and lateral facet (17%) are less injured. Trochlear chondral injuries were evidenced in 27.8% of patients.

In patients with recurrent patellar instability, arthroscopy may be performed as diagnostic and therapeutic tool [35]. Franzone et al. [24] investigated the association between recurrent patellar instability and the location, frequency, and grade of chondral lesions. 57.9% (22/38) of the patients suffering from recurrent dislocations presented advanced chondral lesions in the patella, mostly located on the medial patellar facet [24]. Boddula et al. [22] reported that 45% (9/20) of patients with recurrent patellar instability had chondral lesions. Moreover, concomitant chondral lesions were also associated with lower values in patients reported outcome measures (PROMs) and early onset osteoarthritis [22].

Several surgical strategies are available to manage chondral defects. Microfractures (Mxs) are indicated for smaller defects [36, 37]. Autologous chondrocyte implantation (ACI) has been also widely used for larger chondral defects [38, 39]. However, ACI requires a chondrocyte harvest site, two surgical sessions, and cells expansion in a dedicated laboratory [40, 41]. These features lead to longer recovery, increasing morbidity and the health care burden [42, 43]. To overcome these limitations, autologous matrix-induced chondrogenesis (AMIC) has been introduced [44, 45]. AMIC exploits the regenerative potential of autologous bone marrow derived mesenchymal stem cells, and could be performed in a minimally invasive fashion [46, 47]. For chondral defect of the patella, isolated AMIC performed better compared to isolated MFx [14]. AMIC demonstrated greater International Knee Document Committee (IKDC) and Lysholm Knee Scoring Scale, along with a considerable reduction of the visual analogue scale (VAS), and an earlier return to sport. Furthermore, AMIC demonstrated a lower rate of failure compared to MFx [14].

Patients suffering from recurrent patellofemoral instability present underlying pathoanatomical abnormalities which predispose to dislocation: trochlear or patellar dysplasia, lower limb mal-alignment syndromes such as tibial extra-rotation or femoral anteversion, and soft tissue abnormalities such as patella alta [2, 6, 48–50]. Moreover, most patients present a combination of two or more concomitant pathoanatomical risk factors which synergistically predispose to instability [51, 52]. Thus, the management of recurrent patellofemoral instability can be challenging [53–55]. An adequate evaluation of pathoanatomical risk factors is mandatory to select the proper treatment [56]. The current literature accounts more than thousand articles concerning the management of the patellofemoral instability, but the optimal treatment is still controversial [4, 35]. Conservative strategies are usually preferred as first line management for patellar dislocation [57–60]. However, following conservative management, between 15 and 48% of patients experienced a further patellar dislocation [61]. Surgery is deserved for patients with unstable osteochondral defects or free bodies in the joint cavity, or for patients with recurrent dislocations who have failed conservative management [61].

The present investigation has several limitations. Most of the studies were retrospective, and blinding was seldom performed. The cohort size was limited by most of studies. Most of the included studies did not primarily investigate the rate of chondral injuries, which could represent an important source of bias. Furthermore, relevant patient biometrics characteristics which may influence the patellofemoral biomechanics, such as patella alta, dysplasia, mal-alignment syndromes, and leg axis deformities, were seldom reported. The relatively short length of the mean follow-up by most of studies may jeopardize the efficacy to detect further chondral lesions in the long term. Given these limitations, results of the present systematic review should be interpreted with caution. Future high-quality investigations should validate these findings on a larger scale.

## Conclusion

Chondral defects of the medial facet and the crest of the patella are the most common in patients with recurrent patellofemoral instability.

## Data Availability

No new data were generated or analyzed in support of this review.

## Abbreviations

PRISMA: Preferred reporting items for systematic reviews and meta-analyses
ICRS: International Cartilage Repair Society
NOS: Newcastle–Ottawa Scale
Mxs: Microfractures
ACI: Autologous chondrocyte implantation
AMIC: Autologous matrix-induced chondrogenesis
IKDC: International Knee Document Committee
VAS: Visual analogue scale
